# Protein Kinase CK2 in Cancer Energetics

**DOI:** 10.3389/fonc.2020.00893

**Published:** 2020-06-18

**Authors:** Eduardo Silva-Pavez, Julio C. Tapia

**Affiliations:** Programa de Biología Celular y Molecular, Instituto de Ciencias Biomédicas, Facultad de Medicina, Universidad de Chile, Santiago, Chile

**Keywords:** casein kinase CK2, warburg effect, metabolic switch, aerobic glycolysis, mitochondrial function, hypoxia, autophagy

## Abstract

Protein kinase CK2 (formerly known as casein kinase 2) is abnormally elevated in many cancers. This may increase tumor aggressiveness through CK2-dependent phosphorylation of key proteins in several signaling pathways. In this work, we have compiled evidence from the literature to suggest that CK2 also modulates a metabolic switch characteristic of cancer cells that enhances resistance to death, due to either drugs or to a microenvironment deficient in oxygen or nutrients. Concurrently, CK2 may help to preserve mitochondrial activity in a PTEN-dependent manner. PTEN, widely recognized as a tumor suppressor, is another CK2 substrate in the PI3K/Akt signaling pathway that promotes cancer viability and aerobic glycolysis. Given that CK2 can regulate Akt as well as two of its main effectors, namely mTORC1 and β-catenin, we comprehensively describe how CK2 may modulate cancer energetics by regulating expression of key targets and downstream processes, such as HIF-1 and autophagy, respectively. Thus, the specific inhibition of CK2 may lead to a catastrophic death of cancer cells, which could become a feasible therapeutic strategy to beat this devastating disease. In fact, ATP-competitive inhibitors, synthetic peptides and antisense oligonucleotides have been designed as CK2 inhibitors, some of them used in preclinical models of cancer, of which TBB and silmitasertib are widely known. We will finish by discussing a hypothetical scenario in which cancer cells are “addicted” to CK2; i.e., in which many proteins that regulate signaling pathways and metabolism-linked processes are highly dependent on this kinase.

## Highlights

- Modulation of the Warburg effect and mitochondrial activity.
- Involvement in an Akt and β-catenin-associated metabolic switch.
- Modulation of cancer energetics through autophagy.
- Functional interaction with β-catenin and HIF-1α.

## Introduction

Protein kinase CK2 (formerly known as casein kinase 2) is a constitutively-active kinase that is expressed ubiquitously in eukaryotes (1–3). This butterfly-shaped enzyme is formed by catalytic (α or α') and regulatory (β) subunits and phosphorylates serine o threonine residues within an acidic context (S/TXXD/E/pS/pT/pY), as found in hundreds of proteins in various subcellular compartments, signaling pathways, survival and metabolism-linked processes. CK2 has been shown to be critical in embryonic development, differentiation, immunity, cell survival, epithelial homeostasis and circadian rhythms (4–7). CK2 is also involved in the etiology of many diseases such as multiple sclerosis, cystic fibrosis, chronic intestinal inflammation, cardiac hypertrophy, atherosclerosis, thrombosis, diabetes mellitus, neurological and psychiatric disorders (7–11). In cancer, although CK2 by itself is not an oncogene, some studies have confirmed the tumorigenic potential of this kinase by regulating cellular processes that are characteristic of malignant transformation such as cell cycle progression, tumor growth and death resistance (12). CK2 has been implicated in the regulation of proteins and survival pathways that support chemoresistance, for example, by acquisition of a multi-drug resistance (MDR) phenotype, favoring drug efflux and DNA repair mechanisms (13). Recently, CK2 has been also shown to regulate expression of stemness genes, surface markers and ATP-dependent pumps, accounting for promotion of a stem-like phenotype in colorectal cancer cells (14).

CK2 mRNA levels have been shown to be increased in cancer cells, suggesting that transcriptional mechanisms may play a role in the increase in their protein levels (15). However, post-transcriptional and post-translational mechanisms may also be involved (15–18). Elevated levels of CK2 can be taken as an aggressiveness biomarker, especially the catalytic α subunit, which has been associated to poor prognosis in hepatocellular carcinoma, also correlating with metastatic risk in breast cancer (19, 20). In addition, nuclear localization of CK2α correlates with poor prognosis in renal, prostate and colorectal cancer (21–23), while nuclear localization of CK2β is a marker for predicting outcome of patients with gastric carcinoma (24). In line with this, CK2 has been raised as an attractive therapeutic target for treatment of solid tumors and hematologic malignancies with different kinase inhibitors, including ATP-competitive inhibitors, synthetic peptides and antisense oligonucleotides in preclinical models (25). Moreover, different CK2 inhibitors targeting the catalytic site have been designed, such as 4,5,6,7-tetrabromobenzotriazole (TBB) and silmitasertib (formerly CX-4945) (26). Silmitasertib and CIGB-300, a cell-permeable peptide inhibitor of CK2 (25, 27), have been used in several clinical trials for the treatment of different human cancers (www.clinicaltrials.gov). Nevertheless, CK2 catalyzes the phosphorylation of more than 300 substrates, defining it as the second most pleiotropic member of the human kinome (26, 28), and it also modulates several signal transduction pathways (29). Thus, this apparent pleiotropy must be taken into account before CK2 inhibitors are used to treat cancer or other diseases. Pharmacological inhibition of CK2 may cause unexpected effects, for instance, widespread alterations in alternative splicing of a wide number of genes or inhibition of Cdc2-like kinases, as indeed has been reported elsewhere (28, 30, 31).

More light on the latter could be shed by microRNA studies. Several miRNAs have been reported to downregulate CK2 expression. For example, co-overexpression of miR-760, miR-186, miR-337-3p, and miR-216b decreases CK2α protein levels in IMR-90 human lung fibroblast cells (16). These miRNAs are capable of binding to the 3'-UTR of CK2α mRNA and, consequently, to inhibit its protein expression (16). On the other hand, inhibition of CK2 by quinalizarin in 3T3-L1 pre-adipocyte cells increased miR-27a and miR-27b levels, which target the mRNA of PPARγ, a protein involved in regulation of fatty acid storage and glucose metabolism (32, 33). Also, miR-125b levels have been shown to be significantly decreased in breast cancer (18). This miRNA binds to the 3'-UTR of CK2α mRNA, leading to its decreased expression (18). Furthermore, inhibition of CK2 activity with TBB decreases cell viability and proliferation in MCF-7 breast cancer cells, which correlates with changes in different miRNAs (34). Likewise, CK2β knockdown leads to downregulation of different miRNAs related to cellular processes such as EMT and invasion in MCF10A breast epithelial cells (35). Nevertheless, whether the CK2-related miRNAs are successful in modulating metabolism and bioenergetics in cancer cells remains unknown.

Finally, a growing tumor has a high demand for energy and metabolites necessary for macromolecule biosynthesis. Cancer cells obtain energy mainly from aerobic glycolysis but generate lactate as the final product. This metabolic switch, known as the Warburg effect, is a widely accepted hallmark of cancer (36); however, recent studies indicate that cancer cells may also fully oxidize glucose, which suggests that mitochondrial function is crucial for oncogenesis and progression (37). In any case, either the Warburg effect or mitochondrial function is modulated by the activity of signaling proteins, providing adaptive advantages against a continuously-changing microenvironment. In this review, we compile evidence from the literature suggesting a plausible role for CK2 in modulating several processes related to the energetic changes occurring in a cancer cell, which may ultimately drive a metabolic switch that enhances malignant progression.

## Modulation of Mitochondrial Function

CK2 has been proposed to modulate the Warburg effect in colorectal, esophageal and bladder cancer cells (Table 1). The presence of CK2 increases lactate dehydrogenase A (LDHA) expression and activity as well as proliferation in some of these cells (38–40). This CK2-dependent metabolic switch also promotes *in vitro* invasiveness, partly due to the regulated differential expression of two pyruvate kinase isoforms, PKM1 and PKM2 (39). The constitutively-active PKM1 isoform is down-regulated in cells overexpressing CK2, while the PKM2 isoform is imported into the nucleus (39). PKM2 is a cofactor of hypoxia-inducible factor-1 (HIF-1), whose transcriptional targets are LDHA, glucose transporter 1 (GLUT1), and pyruvate dehydrogenase kinase 1 (PDK1) (64). Of note, both pharmacological inhibition and siRNA-mediated silencing of CK2 lead to inhibition of the Warburg effect observed in bladder cancer cells (40).

**Table 1:** Effect of CK2 activity alterations on both mitochondrial- and energetics-related components in several cancers.

**CK2 alterations**	**Effects**	**Cancers**	**References**
Overexpression	Increment of LDHA expression and activity, down-regulation of PKM1 isoform and nuclear import of PKM2 isoform.	Colorectal, esophagus, bladder	(38–40)
Overexpression	Increased glucose consumption and extracellular lactate levels, which is blocked by inhibition of LDHA.	Colorectal	(38, 39)
siRNA silencing	Inhibition of the Warburg effect.	Bladder	(40)
Inhibition (TBB)	Mitochondrial membrane depolarization.	Prostate	(41)
NE	β-catenin-dependent increased expression of MCT-1 and PDK1.	Colorectal	(42, 43)
Overexpression	β-catenin-dependent increased expression of survivin.	Colorectal	(44–46)
NE	Survivin increases the Warburg effect through mitochondrial complex II stability.	Colorectal	(47)
NE	β-catenin-dependent increased expression of c-Myc, ASCT2, and glutaminase.	Colorectal, breast	(48)
NE^a^	p27, p62, and probably ULK-1, are substrates of CK2.	Colorectal	(49–51)
Inhibition (TBB, quinalizarin)	ATF4-regulated expression of proteins at autophagy, amino acid biosynthesis and transport, lipid and glucose metabolisms.	Colorectal	(52–57)
Inhibition (silmitasertib)	Reduction of mTORC1 activity.	Colorectal, squamous, lung	(51, 58, 59)
Inhibition (TBB, siRNA)	HIF1α-regulated expression of aldolase and p53.	Hepatocellular, cervical	(60–63)

*NE, not experimentally demonstrated for this cancer*.

a *only suggested for ULK1*.

Mitochondrial function is essential to the metabolic switch that is characteristic of cancer (Table 1). Qaiser et al. showed that CK2 may help to preserve mitochondrial activity in prostate cancer cells. They found CK2 enriched in mitochondria from several prostate cancer cell lines, somehow supporting membrane polarity, which is also essential for the electron transport chain. Thus, pharmacological inhibition of CK2 may generate rapid membrane depolarization just before the onset of apoptosis (41). This effect may be dependent on the tumor suppressor phosphatase and tensin homolog (PTEN), which is also phosphorylated by CK2, promoting its stability and cytoplasmic enrichment (65).

Indeed, phospho-PTEN mimics the tumorigenic effects observed upon deletion or mutant inactivation of its coding gene (66). Expression of a long PTEN isoform (PTENα) has been observed in prostate cancer cells with loss of PTEN function. This isoform is generated by alternative translation at a non-canonical CUG initiation site in the 5'UTR. PTENα is mainly located in the mitochondria and interacts with normal PTEN. Together, these isoforms stabilize PTEN-induced kinase 1 (PINK1), a serine/threonine kinase associated with degradation of dysfunctional mitochondria (67). Interestingly, ectopic PTENα expression in PTEN-null cell lines leads to increased mitochondrial function accompanied by elevated ATP production and cytochrome c oxidase activity (68). In this alternative translation of PTEN, recognition of the start codon is strongly regulated by the stoichiometry of various eukaryotic initiation factors (eIF) that form the pre-initiation complex (PIC) along with other proteins. CK2 and the mammalian target of rapamycin complex 1 (mTORC1) coordinate PIC assembly, promoting proliferation upon stimulation with growth factors and nutrients (69). Here, the two kinases activate the translation process by phosphorylation of eIF2β. Of note, CK2-mediated phosphorylation of eIF5 has been deemed important for cell cycle progression (70); however, recognition of CUG at the PTENα 5'UTR is mediated by eIF2α (68). Therefore, whether eIF2α is a target of CK2 or has a role in the PTENα/PTEN complex in supporting PINK1 stabilization at the mitochondrial membrane remains entirely unknown.

## PI3K/AKT and β-Catenin-Related Metabolic Switch

PTEN is a widely-known tumor suppressor in the PI3K/Akt signaling pathway (Table 1), which plays a key role in cancer due to its relationship with cellular processes involved in proliferation, apoptosis, and invasiveness, as well as energetics (71). Akt activation is achieved by phosphorylation at Thr-308 by the phosphoinositide-dependent kinase 1 (PDK-1) and at Ser-473 by mTORC2 (mTOR complex 2). CK2 phosphorylates Akt at Ser-129, a residue located in a linking region between the PH and catalytic domains, which stabilizes and increases β-catenin activity (44), suggesting an important role for CK2 in regulating cancer energetics and malignant progression of several cancers. Once fully activated, Akt dissociates from the membrane and phosphorylates various proteins, including tuberous sclerosis complex 1/2 (TSC1/2) in the PI3K/Akt/mTORC1 signaling pathway (72).

Akt also phosphorylates β-catenin at Ser-552 (73), an essential component of the canonical Wnt signaling pathway (Table 1). Thus, Akt may be sufficient to promote both the metabolic switch and proliferation of several types of cancer cells (71). The canonical Wnt signaling pathway is involved in cell proliferation, migration, and other events traditionally considered to be hallmarks of cancer. In unstimulated cells, β-catenin is down-regulated by a multiproteic complex formed by Axin, GSK-3β, and the tumor suppressor APC. Axin facilitates the phosphorylation of β-catenin by GSK-3β at specific serine and threonine residues at its N-terminal end, driving β-catenin to its ubiquitination and degradation by the proteasome (74, 75). Conversely, aberrant activation of the canonical Wnt pathway in cancer leads to β-catenin stabilization, nuclear translocation, and interaction with the TCF/LEF family of transcription factors (76). Nuclear β-catenin thus drives expression of proteins such as c-Myc, cyclin-D1, cyclooxygenase-2 (COX-2), and survivin (45, 74, 77), which are primarily related to proliferation, apoptosis resistance, and metastasis, as well as other proteins such as monocarboxylate transporter-1 (MCT-1) and pyruvate dehydrogenase kinase-1 (PDK1), which are associated to cancer energetics (42, 43). Interestingly, the β-catenin antagonist Chibby is related to inhibition of the metabolic switch observed in nasopharyngeal carcinoma (78).

Canonical Wnt pathway activity is increased when β-catenin is phosphorylated at Ser-552 by the Akt kinase (73). Moreover, Akt is phosphorylated by CK2 at Ser-129, which stabilizes and increases β-catenin activity (44), strongly suggesting an important role for CK2 in regulating cancer energetics and malignant progression of several cancers. In addition to Akt, CK2 can also directly up-regulate β-catenin activity by phosphorylating it at Thr-393, which should impede its binding to Axin and APC. Disruption of this binding would block proteasomal degradation, increase stability, and boost nuclear activity of β-catenin (79–82). Several findings have demonstrated that the catalytic CK2α subunit indeed activates the canonical Wnt pathway. This subunit increases β-catenin, COX-2, and survivin expression at the transcriptional level, promoting proliferation and apoptosis resistance in colorectal cancer cells (44, 46, 83). Of note, survivin increases the Warburg effect in PC3 prostate cancer cells by increasing mitochondrial complex II stability (47). Likewise, increased survivin levels correlate with enhanced aerobic glycolysis attributable to mitochondrial function regulation in neuroblastoma cells (84).

Another target of CK2 and β-catenin, oncogenic c-Myc, also has a role in the Warburg effect by inducing the expression of genes related to glucose-derived energetics and glutamine-dependent metabolism, such as the glutamine transporter (ASCT2) and the enzyme glutaminase (48). Interestingly, in colorectal and breast cancer cells, the canonical Wnt pathway may contribute to the maintenance of stemness by regulating the metabolic switch in cancer stem cells (48). Catalytic CK2α subunit overexpression may also increase glucose consumption and extracellular lactate levels in colorectal cancer cells (39). Besides, glucose (but not glutamine) is necessary for the maintenance of CK2α-dependent viability, migration, and invasion in these cells, while those properties are blocked upon inhibition of LDHA (38). This strongly suggests that reduction of migration and invasion via LDHA inhibition could be used as a potential therapy for CK2α-dependent tumors.

Upon silencing of β-catenin expression in breast cancer cells, levels of proteins involved in glucose metabolism and the tricarboxylic acid (TCA) cycle are decreased, while levels of proteins associated with lipid metabolism are increased (85). Additionally, β-catenin silencing promotes the use of acetate while decreasing use of glucose for fatty acid synthesis (85). Finally, β-catenin silencing in breast cancer cells decreases mRNA levels of the peroxisome proliferator-activated receptor gamma coactivator 1-α (PGC-1α), mitochondrial transcription factor A (TFAM), nuclear respiratory factor-1 (Nrf1), and GLUT-1, thus increasing levels of acetyl-CoA carboxylase (ACC), fatty acid synthase (FASN), and sterol regulatory element-binding protein 1 (Srebp1) (85). Interestingly, activation of EGFR induces translocation of the PKM2 enzyme to the nucleus, where it interacts with β-catenin and thereby increases c-Myc expression (86).

## Modulation of Cancer Energetics by Autophagy

A key component of the PI3K/Akt/mTORC1 signaling pathway is the mTOR subunit (Table 1), a Ser/Thr protein kinase frequently deregulated in cancer (87, 88). As with the C1 complex, mTOR regulates processes involved in growth-associated metabolism such as protein synthesis through phosphorylation of the effector S6 kinase 1 (S6K1), which promotes ribosomal translation by phosphorylating the ribosomal protein S6 (88). MTORC1 also stimulates translation of the mitochondrial fission process 1 (MTFP1) protein, which controls mitochondrial fission and apoptosis (89). Mitochondrial function is also modulated by mTORC1 through regulation of TFAM levels, promoting mitochondrial DNA replication, transcription, and mitochondrial biogenesis (90). In addition, mTORC1 is crucial in autophagy, a catabolic process of cellular response to low levels of nutrients and growth factors, in which lysosomal enzymes degrade intracellular components and molecules to maintain energetic homeostasis and viability (88).

Despite the above, the role of autophagy as an oncogenic factor or tumor suppressor is controversial and may depend on the origin and progression of the tumor (91). The molecular mechanism for regulating autophagy involves various proteins, including mTORC1, which is down-regulated by the TSC1/2 complex but up-regulated by the Akt kinase, which phosphorylates and inactivates TSC1/2 (71). Consequently, TSC1/2 inactivation favors activation of Rheb, a small GTPase that induces autophagy through both p27/Kip1- and mTORC1-dependent mechanisms. The cell-cycle inhibitor p27/Kip1 has been shown to have a key role in the cellular effect of Rheb in response to serum deprivation in colorectal cancer cells (92). Rheb interacts with and activates mTORC1, which then phosphorylates ULK-1, a kinase responsible for triggering autophagic flux, which is characterized by decreased p62 levels (87, 88, 93). Interestingly, both p27 (49) and p62 (50), and probably also ULK-1 (51), are proteins phosphorylated by CK2.

Genetic and epigenetic alterations in some components of the PI3K/Akt/mTORC1 pathway have been described, such as activating mutations in oncogenes PI3KCA (94) and mTOR (95), loss of function of tumor suppressor PTEN (96), and overexpression of oncogene Akt (97). All of these alterations contribute to an aberrant activation of the pathway, leading to increased tumor growth and ultimately a metastatic phenotype (98). CK2 phosphorylates Akt at Ser-129, and the mutation of this residue to alanine causes a marked decrease in Akt activity. Furthermore, pharmacological inhibition of CK2 or siRNA-mediated reduction of CK2α diminish Akt activity, which is independent of its phosphorylation at Thr-308 and Ser-473 (99). In addition, phosphorylation at Ser-129 has been suggested to play a key role in promoting proliferation of colorectal cancer cells in a β-catenin-dependent way (44), but also in glioblastoma and lung cancer cells through mTORC1 activation (58, 100).

Evidence in the literature suggests that CK2 modulates mTORC1 activity, and decreasing CK2α expression leads to increased autophagy-dependent cell death (ADCD) in glioblastoma cells, which correlates with decreased phosphorylation of S6K1 and Akt (100). Interestingly, expression of proteins that participate in autophagy, amino acid biosynthesis and transport, lipid and glucose metabolism are regulated by Activating Transcription Factor 4 (ATF4) (52, 53). Indeed, CK2 neutralizes the function of ATF4 by phosphorylation; however, under CK2 inhibition, protein levels and transcriptional activity of ATF4 increase (54–56). Moreover, ATF4 promotes the expression of the transcriptional C/EBP Homologous Protein (CHOP) factor, which induces apoptosis via ER stress signaling in colon cancer cells (55, 57). Of note, ATF4 participates, together with the protein kinase RNA-like endoplasmic reticulum kinase (PERK) and eIF2α, in the formation of respiratory chain supercomplexes by increasing levels of SR-related CTD-associated factor 1 (SCAF1), consequently enhancing mitochondrial respiration (101).

**Figure 1:**
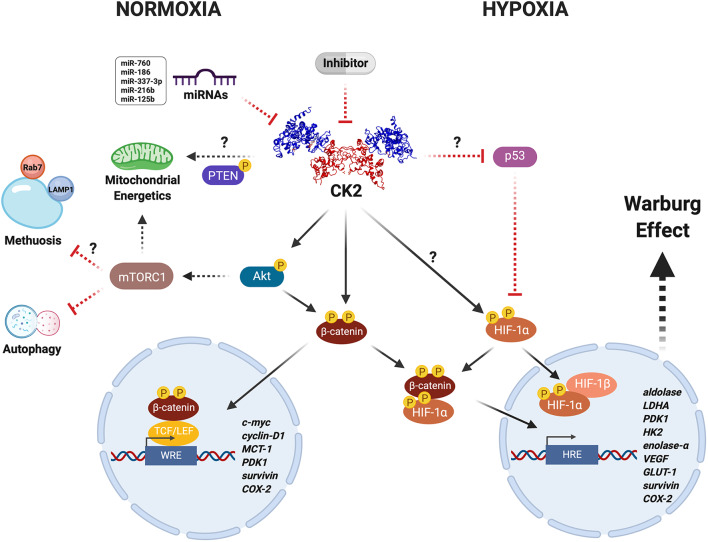
CK2 modulates the Warburg effect in cancer cells under unfavorable conditions. CK2 (PDB: 1JWH) may have a central role in regulating the activity and stability of various proteins including Akt, PTEN, β-catenin, and HIF-1α, under physiological and pathological conditions. CK2-dependent activation of the Wnt/β-catenin, PI3K/Akt/mTORC1, and p53/HIF-1α signaling pathways in cancer cells leads to a metabolic switch that supports proliferation and resistance to death due to either neoplastic drugs or an oxygen- and nutrient-deficient microenvironment, as a result of increased expression of genes that collectively enhance mitochondrial function and glucose metabolism. Altogether, this may be heavily altered upon inhibition with very specific compounds like the CK2 inhibitor, silmitasertib, or alternatively some miRNAs (more details in text). However, whether the latter are able to modulate metabolism and bioenergetics in cancer cells remains yet unknown.

In addition, CK2 inhibition with silmitasertib induces autophagy-triggered apoptosis when used alone in rat and human chondrocytes (102). Silmitasertib treatment correlates with decreased mTORC1 activity and massive formation of large acidic LC3-negative cytoplasmic vacuoles in colorectal cancer cells (Figure 1). However, while there has been no significant evidence of enhanced autophagy in the presence of silmitasertib, studies have shown elevated levels of a macropinocytosis-linked cell death known as methuosis after silmitasertib treatment (51). In this context, modest levels of autophagy and macropinocytosis should coexist to promote survival in adverse energetic conditions (i.e., unfavorable levels of nutrients, oxygen, etc.); however, CK2 inhibition may cause a shift toward aberrant macropinosome formation, ultimately leading to increased cell death. On the other hand, silmitasertib combined with the EGFR inhibitor erlotinib produces a complete inhibition of the PI3K/Akt/mTORC1 pathway, inducing apoptosis in squamous carcinoma and lung cancer cells (58). Moreover, a combination of silmitasertib and another EGFR inhibitor, gefitinib, decreases proliferation and induces apoptosis in lung cancer cells (59).

## A Harmful Linkage With β-Catenin and HIF-1α

Tumors progress in a hypoxic or low-oxygen microenvironment. In this cellular context, the protein HIF-1 plays an essential role in the metabolic switch that promotes the survival of cancer cells (103, 104). HIF-1 is a transcription factor formed by two subunits, HIF-1α, whose expression is inducible, and HIF-1β, also known as aryl hydrocarbon receptor nuclear translocator (ARNT), whose expression is constitutive. In the presence of oxygen, the HIF-1α subunit is cytosolically hydroxylated at prolines, allowing for recruitment of a ubiquitin-ligase complex that contains the Von Hippel-Lindau (VHL) tumor suppressor protein, promoting HIF-1α degradation. Under hypoxia, enhanced HIF-1 stability and activity help tumor cells to survive (105). Stable HIF-1α translocates to the nucleus and interacts with HIF-1β for binding to hypoxic response elements (HRE) at promoter sequences, inducing expression of genes linked to the metabolic switch and other hallmarks of cancer, such as aldolase, LDHA, PDK1, hexokinase 2 (HK2), enolase-α, VEGF, GLUT-1, survivin, and COX-2 (104, 106). Consequently, VHL inactivation also increases the stability of HIF-1α, its nuclear translocation, and expression of target genes (107). For example, over 90% of renal cell carcinomas (RCC) harbor a biallelic inactivation of the VHL gene, becoming highly dependent on aerobic glycolysis for ATP production. Thus, pharmacological impairment of glucose transport results in specific death of RCCs (107, 108).

CK2 activity and levels are elevated in hepatocellular and cervical cancer cells grown under hypoxia, concomitant with increased nuclear localization (60, 61). Interestingly, CK2 inhibition with TBB, 5,6-dichloro-1-β-D-ribofuranosylbenzimidazole (DRB), or apigenin, as well as overexpression of a dominant negative form of CK2α or siRNA-mediated silencing, are all capable of decreasing the transcriptional activity of HIF-1 without altering its protein levels or HRE-binding capacity (60). However, other CK2 inhibitors, such as E9 and silmitasertib, induce a significant decrease in HIF-1α levels in the same cells (62). In addition, CK2 decreases the stability of the tumor suppressor VHL in embryonic kidney cells, while its inhibition with TBB leads to its stabilization, triggering HIF-1 degradation and thereby diminished HRE-associated transcriptional activity (109). These findings strongly suggest an important role for CK2 in regulating HIF-1 stability and activity (Figure 1).

Low levels of oxygen inhibit the canonical Wnt pathway in colorectal cancer cells, thereby decreasing expression of its target genes. Here, HIF-1α may be able to interact with β-catenin, preventing its interaction with Tcf-4 (105). Likewise, the HIF-1α/β-catenin complex may bind to the HRE of target genes. Moreover, β-catenin silencing significantly decreases the viability of colorectal cancer cells, likely through inhibition of HIF-1-dependent transcriptional activity (105). On the other hand, COX-2 expression is increased under hypoxia in colorectal cancer cells. The binding of HIF-1α to the HRE of the COX-2 promoter has been observed under these conditions, increasing prostaglandin E2 (PGE2) synthesis and favoring cell proliferation (110). Similar β-catenin-dependent regulated COX-2 expression and PGE2 synthesis have been observed in colorectal and breast cancer cells, as well as in embryonic kidney cells growing under normal oxygen levels, where CK2 expression was either up- or down-regulated (83). While expression of HRE target genes associated with the metabolic switch was not assessed in this study, a role for the HIF-1α/β-catenin complex cannot be ruled out (Figure 1). In fact, an *in silico* analysis showed that HIF-1α contains five putative phosphorylation sites for CK2, namely Ser-551, Ser-581, Ser-786, Thr-700, and Thr-796 (60). Moreover, pharmacological inhibition of CK2 with DRB and apigenin decreases aldolase mRNA levels and VEGF secretion in hepatocellular cancer cells exposed to hypoxia (60). Likewise, CK2 inhibition in cervical and hepatocellular cancer cells grown under hypoxia drives an increase in the tumor suppressor p53, its interaction with HIF-1α, and blockage of interaction with HIF-1β, thereby inhibiting HRE-dependent transcriptional activity (63).

## Concluding Remarks/Perspectives

CK2 catalyzes the phosphorylation of more than 300 substrates, thereby constituting the second most pleiotropic member of the human kinome (26, 28). Some CK2 protein substrates are crucial in various signaling pathways linked to hallmarks of cancer. Thus, it is easy to understand how this kinase may modulate cancer malignancy. In this respect, CK2 is thought of as a non-oncogene target to which some cancers may become “addicted,” as proposed early on by Ruzzene and Pinna (29). We have here compiled evidence from the literature suggesting an important role for CK2 in the capacity of some cancer cells to undergo a metabolic switch that confers resistance to death by therapeutic drugs or in response to an unfavorable microenvironment. It is possible that CK2 inhibition may be catastrophic for cancer cells addicted to CK2, leading to massive cell death directly or indirectly perhaps by modulating the function of key targets responsible for the Warburg effect, such as β-catenin or HIF-1, or even by regulating mitochondrial activity via PTEN or autophagy via mTORC1 (Figure 1). Something like this seems to happen in colorectal cancer cells treated with the CK2 inhibitor silmitasertib. These cells fall into an irreversible process of self-destruction known as methuosis, a massive entry of extracellular material by macropinocytosis (51).

In a scenario in which cells are addicted to CK2, many proteins that modulate signaling pathways might be highly dependent upon this kinase, along with other key factors modulating glucose metabolism. This fact highlights a putative role of CK2 as an aggressiveness biomarker. Indeed, the catalytic α subunit may serve as a poor prognosis factor in liver and breast cancer (19, 20), while nuclear localization of both catalytic α and regulatory β subunits may be independently used for predicting the outcome of patients with gastric, renal, prostate or colorectal cancer (21–23). Consequently, specific CK2 inhibition may lead to a loss of viability and metabolic catastrophe in cancer cells. The ubiquitous expression of CK2 and its role in many physiological processes raise some doubts concerning its feasibility as a therapeutic target. However, inhibition of CK2 with silmitasertib, when combined with other drugs such as cisplatin, paclitaxel, temozolomide, gemcitabine or gefitinib, among others, has shown synergistic effects in preclinical models of cancer by decreasing tumor growth (13). Moreover, addiction to CK2 would seem to make cancer cells very susceptible to highly specific inhibitors. In fact, several preclinical studies of newly specific CK2 inhibitors have yielded very promising results. Thus, inhibition of CK2 in CK2-addicted tumors, i.e., cells with markedly elevated CK2 expression and activity, may offer a real therapeutic opportunity in the future.

## Author Contributions

ES-P and JT designed and outlined the structure, contents of the review, contributed to the literature review, discussion, and writing of the manuscript. All authors contributed to manuscript revision and approved the version to be published.

## Conflict of Interest

The authors declare that the research was conducted in the absence of any commercial or financial relationships that could be construed as a potential conflict of interest.
